# Green/Roasted Coffee and Silverskin Extracts Inhibit Sugar Absorption by Human Intestinal Epithelial (Caco-2) Cells by Decreasing GLUT2 Gene Expression

**DOI:** 10.3390/foods11233902

**Published:** 2022-12-03

**Authors:** Juliana A. Barreto Peixoto, Nelson Andrade, Susana Machado, Anabela S. G. Costa, Maria Beatriz P. P. Oliveira, Fátima Martel, Rita C. Alves

**Affiliations:** 1 REQUIMTE/LAQV, Department of Chemical Sciences, Faculty of Pharmacy, University of Porto, R. Jorge Viterbo Ferreira 228, 4050-313 Porto, Portugal; 2 Unit of Biochemistry, Department of Biomedicine, Faculty of Medicine, University of Porto, Al. Prof. Hernâni Monteiro, 4200-319 Porto, Portugal; 3 Instituto de Investigação e Inovação em Saúde (I3S), University of Porto, R. Alfredo Allen 208, 4200-135 Porto, Portugal

**Keywords:** coffee, by-product, ultrasound extraction, 5-caffeoylquinic acid, caffeine, chromatography, Caco-2 cells, sugar uptake, glucose, fructose, gene expression

## Abstract

Moderate coffee ingestion has been associated with a decrease in type 2 diabetes risk, mainly due to its richness in chlorogenic acids (CGA). To explore this, extracts of green beans, roasted beans, and silverskin were prepared by aqueous ultrasound-assisted extraction and characterized by a reversed-phase high-performance liquid chromatography-photodiode array detector (RP-HPLC-DAD). The effects on the uptake of glucose and fructose by human intestinal epithelial (Caco-2) cells and the influence on the expression of sugar transporter genes (by RT-qPCR) were investigated and compared. The uptake of ^3^H-deoxy-D-glucose and ^14^C-fructose by Caco-2 cells was significantly reduced by all the extracts, with green coffee (which also contained higher amounts of CGA) achieving the highest efficiency. Although silverskin presented the lowest amounts of CGA and caffeine, it promoted an inhibitory effect similar to the effects of green/roasted beans. In the case of glucose uptake, the effect was even higher than for roasted coffee. This activity is explained by the ability of the extracts to markedly decrease GLUT2, but not GLUT5 gene expression. In addition, a decrease in SGLT1 gene expression was also found for all extracts, although not at a statistically significant rate for silverskin. This study also revealed a synergistic inhibitory effect of caffeine and 5-CQA on the uptake of sugars. Thus, silverskin appears as an interesting alternative to coffee, since the valorization of this by-product also contributes to the sustainability of the coffee chain.

## 1. Introduction

Coffee is one of the most appreciated beverages worldwide, not only due to its pleasant flavor and sensory properties, but also due to its increasing association with several potential health benefits, such as decreasing the risk of type 2 diabetes; cancer; depression; cardiovascular, neurodegenerative, and hepatic diseases; as well as improvement in gut microbiota and antioxidant defenses [1,2,3,4,5]. Most of these properties have been widely attributed to chlorogenic acids (CGA), caffeine, trigonelline, dietary fiber, and melanoidins [1,3,6].

The high level of coffee production (175 million 60 kg bags in 2020 [7]) required to satisfy consumer demand also generates significant amounts of coffee by-products along the chain, which represents a serious environmental concern. In this context, in recent years, several studies have been conducted to valorize these different coffee by-products (wastes) generated, as they are also a huge source of several bioactive compounds and, hence, might exhibit potential health benefits and may be used in the development of functional food products [8]. Particularly, coffee silverskin (a thin tegument that detaches from the beans during roasting due to their heat-induced expansion), which represents the main by-product of the coffee roasting industries, has been described as an important source of dietary fiber (mainly insoluble) and antioxidants, such as phenolics (mainly CGA), caffeine, and melanoidins [9,10,11]. Therefore, considering the aforementioned beneficial effects associated with these compounds, coffee silverskin might be a useful ingredient for the development of functional products.

It has been widely recognized that moderate coffee consumption is positively related to a decrease in the risk of developing type 2 diabetes, especially due to its richness in CGA [1,3,12]. The same assumptions have been suggested for coffee silverskin, based on its chemical composition [13]. Importantly, high sugar intake—particularly from high glycemic foods and foods with added sugars containing fructose (including sucrose and high-fructose corn syrup (HFCS)—has been associated with the development of type 2 diabetes and metabolic syndrome [14,15,16]. Furthermore, several studies have also demonstrated that by reducing the intestinal uptake of glucose and fructose, the metabolic consequences of their ingestion can be also reduced, thus decreasing the risk of metabolic syndrome [12,17].

In this context, it was previously noted that some CGA present in coffee are able to inhibit glucose intestinal transport and absorption [18,19,20,21,22,23], but little is yet known about their effects on fructose intestinal uptake.

Supported by this fact, the present work aimed to compare, for the first time, as far as we know, the effects of green coffee, roasted coffee, and coffee silverskin extracts on the uptake of two sugars (glucose and fructose) by human intestinal epithelial (Caco-2) cells—a cell model widely used to mimic the intestinal absorption of sugars [24,25]—as well as on the expression levels of different intestinal sugar transporters. Moreover, a possible correlation of the chemical composition of each extract (analyzed by RP-HPLC-DAD) with the observed biological effects was also explored. In order to avoid the external influence of different chemical compositions related to distinct coffee species or geographical origins, this study used only one type of coffee beans—green beans from the robusta species (*Coffee canephora*) (chosen due to its recognized richness in caffeine and CGA, compared to arabica coffee [6])—from a selected geographical origin (Cameroon, Africa). These beans were then roasted to obtain roasted beans and coffee silverskin.

## 2. Materials and Methods

### 2.1. Reagents and Standards

Quantities of 5-, 4-, and 3-caffeoylquinic acids (5-CQA, 4-CQA, 3-CQA), 5-, 4- and 3-feruloylquinic acids (5-FQA, 4-FQA, 3-FQA), caffeine, glacial acetic acid, and methanol (HPLC grade), were purchased from Sigma-Aldrich (St. Louis, MI, USA).

^14^C-D-Fructose (^14^C-FRU) (250–360 mCi/mmol) and [1,2-3H(N)]-deoxy-D-glucose (^3^H-DG) (60 mCi/mmol) were purchased from American Radiolabeled Chemicals (St. Louis, MO, USA). Antibiotic/antimycotic solution (0.25 mg/mL amphotericin B, 100 mg/mL streptomycin, and 100 U/mL penicillin), MEM medium, N-2-hydroxyethylpiperazine-N′-2-ethanesulfonic acid (HEPES), reduced nicotinamide adenine dinucleotide (NADH), ethylenediamine tetra-acetic acid (trypsin–EDTA), sulforhodamine B (SRB), sodium pyruvate, sodium salt, and trichloroacetic acid were obtained from Sigma. DMSO and Triton X-100 were obtained from Merck (Darmstadt, Germany), and fetal calf serum was purchased from Invitrogen Corporation (Carlsbad, CA, USA). NZYol reagent was purchased from NZYTech (Lisbon, Portugal), qScript cDNA SuperMix was obtained from Quanta Biosciences (Gaithersburg, MD, USA), and KAPA SYBRw FAST quantitative polymerase chain reaction (qPCR) Master Mix came from KAPA SYBRw FAST qPCR Kit Master Mix Universal, Kapa Biosystems (Wilmington, MA, USA).

Ultrapure water was obtained using a Milli-Q water purification system (Millipore, Bedford, MA, USA). All other reagents were of analytical grade quality.

### 2.2. Samples and Sample Preparation

Green beans (~5 kg) from robusta coffee (*Coffea canephora*) produced in Cameroon, Africa, were kindly supplied by a Portuguese coffee company (BICAFÉ—Torrefação e Comércio de Café Lda.). The sample was divided in half, and one part was roasted under a standard industrial roasting protocol (~210 °C, 10 min). After this procedure, the roasted coffee beans and coffee silverskin were collected separately and stored at room temperature in a dry place and protected from light. Before the preparation of the extracts, all samples were ground and homogenized.

### 2.3. Ultrasound-Assisted Extraction

The extracts were prepared by ultrasound-assisted extraction using a HD4050 SONOPULS ultrasonic homogenizer from BANDELIN electronic GmbH & Co., KG, Heinrichstrasse, Germany. The equipment was composed of an ultrasonic probe, an ultrasonic generator, and an ultrasonic converter.

The extracts were prepared using an amount (~0.500 g) of ground sample and 25 mL of distilled water. Each sample was extracted for 10 min, without pulsation, applying a high-frequency energy of 20 kHz (±0.5 kHz), which was converted to mechanical deflections on the tip of the ultrasound probe with a constant amplitude of 50%. In the end, the replicates (*n* = 8) were combined, filtered, and freeze-dried (−80 °C, 0.015 mbar; Cryodos, Telstar, Barcelona, Spain).

### 2.4. RP-HPLC-DAD Analysis

A similar amount (~10 mg) of each freeze-dried extract (previously homogenized) was redissolved in 10 mL of distilled water and centrifuged (Labofuge Ae, Heraeus Sepatech; 4500 rpm, 10 min). Then, a supernatant aliquot was collected into Eppendorf tubes and centrifuged again (Heraeus Fresco 17 Centrifuge, Thermo Fisher Scientific, Schwerte, Germany; 13,000 rpm, 10 min). Next, the new supernatant was transferred to injection vials and analyzed by RP-HPLC-DAD, according to the method of Puga et al. [26], with minor modifications. The chromatographic analysis was performed using an HPLC integrated system (Jasco, Tokio, Japan) equipped with an AS-950 automated injector (20 μL loop), two Jasco PU-2080 Plus HPLC pumps, and an MD-2010 Plus multiwavelength diode array detector. The compounds were separated using a reverse-phase Tracer-Excel ODSA (5 μm; 250 × 4 mm) from Teknokroma (Barcelona, Spain). A gradient solvent system of acetic acid:water (0.2:99.8) (A) and methanol (B) was used as follows: 0 min, 7.5% B; 10 min, 20% B; 12 min, 30% B; 20 min, 35% B; 28 min, 40% B; flow rate: 1 mL/ min. CGA were monitored at 320 nm and caffeine at 274 nm. The identification of compounds was performed by comparing retention times, elution orders, and UV absorption spectra with those in the literature and authentic standards. The results are expressed as μg of compound/g of freeze-dried extract. The analyses were run in triplicate.

### 2.5. Cellular Assays

#### 2.5.1. Caco-2 Cell Culture

The Caco-2 cell line was obtained from American Type Culture Collection (HTB-37; Manassas, VA, USA) and was used between passage numbers 8–18. The cells, maintained in a humidified atmosphere (5% CO_2_ and 95% air), were grown in Minimum Essential Medium containing glucose (5.55 mM), fetal calf serum (15%), HEPES (25 mM), amphotericin B (0.25 μg/mL), streptomycin (100 μg/mL), and penicillin (100 units/mL). The culture medium was replaced every 3–4 days, and the culture was split every 10 days. For sub-culturing, the cells were removed enzymatically (0.25% trypsin-EDTA, 5 min, 37 °C), split 1:3, and sub-cultured in plastic culture dishes (21 cm^2^; ∅ 60 mm; Corning Costar, Corning, NY, USA).

To perform the assays, Caco-2 cells were seeded on 24-well plastic cell culture clusters (2 cm^2^; ∅ 16 mm; Corning Costar) and were used 10 days after the initial seeding (100% confluence, corresponding to an average number of 2 × 10^5^ cells). The culture medium was made free of fetal bovine serum for 24 h before the experiments.

#### 2.5.2. Cell Treatments

A similar amount (~10 mg) of each freeze-dried extract was first dissolved in 100 μL of distilled water (100 mg/mL). Different extract concentrations (0.01, 0.1, and 1 mg/mL for the assays assessing the uptakes of ^3^H-DG and ^14^C-FRU, and 1 mg/mL for the remaining tests) were prepared using culture medium or glucose-free Krebs buffer. For the controls, distilled water was used.

The effects of both standards, caffeine and 5-caffeoylquinic acid (the main compounds found in the samples), separately and together, at the concentrations present in 1 mg/mL of the correspondent sample extract (determined by RP-HPLC-DAD, Section 2.4), on the uptake of ^3^H-DG and ^14^C-FRU by Caco-2 cells, were also evaluated. The standards were dissolved in DMSO, and their controls were run in the presence of this solvent.

None of the solvents used (distilled water or DMSO) influenced the measured parameters.

#### 2.5.3. Evaluation of Uptake of ^3^H-DG and ^14^C-FRU by Caco-2 Cells

The uptake experiments were performed in glucose-free Krebs buffer (containing, in mM: 125 NaCl, 25 NaHCO_3_, 20 HEPES, 4.8 KCl, 1.6 KH_2_PO_4_, 1.2 MgSO_4_, 1.2 CaCl_2_, and 0.4 K_2_HPO_4_ (pH 7.4)). Initially, the culture medium was removed, and the cells washed with buffer at 37 °C (0.3 mL). Then, the cell monolayers were preincubated for 20 min in 0.3 mL buffer at 37 °C. Uptake was then initiated by the addition of 0.3 mL medium at 37 °C containing 10 nM ^3^H-DG or 100 nM ^14^C-FRU. Incubation ended after 6 min by removing the medium, placing the cells on ice, and rinsing them with 0.5 mL ice-cold buffer, which will remove any ^3^H-DG or ^14^C-FRU adsorbed onto the cells or the plastic. Then, the cells were solubilized with 0.3 mL of 0.1% Triton X-100 (in 5 mM Tris HCl, pH 7.4) and left overnight (37 °C). Cell radioactivity was measured by liquid scintillation counting. The extracts or compounds to be tested were present 24 h before the uptake assays and also during preincubation and incubation.

#### 2.5.4. Determination of Cell Viability

After a 24 h of exposure to the extracts, the cell viability was assessed through extracellular LDH activity using the method described by Andrade et al. [27].

#### 2.5.5. Determination of Culture Mass

Sulforhodamine B has the capacity to electrostatically bind to basic amino acids of cell culture proteins previously fixated with TCA (trichloroacetic acid). It measures whole-culture protein content as an index of culture growth. After a 24 h exposure to the extracts, the cell culture mass was determined by the sulforhodamine B (SRB) assay, as described by Andrade et al. [27]. Briefly, after treatment, the cell monolayers are fixed with 10% (*w*/*v*) trichloroacetic acid, and the excess dye is removed by repeatedly washing with 1% (*v*/*v*) acetic acid. The protein-bound dye is solubilized in Tris-NaOH solution and the absorbance at 510 nm is then measured.

#### 2.5.6. Quantitative Reverse Transcription Real-Time PCR

Total RNA was extracted from Caco-2 cells treated for 24 h with the extracts/compounds, using NZYol^®^ isolation reagent (NZYTech, Lisbon, Portugal). Before cDNA synthesis, total RNA was treated with DNAse I, and 1 mg of the resulting DNA-free RNA was reverse transcribed using qScript cDNA SuperMix (Quanta Biosciences, Gaithersburg, MD, USA) in 20 mL of final reaction volume. The procedures were performed according to the manufacturers’ instructions.

A Lightcycler96 (Roche Applied Science, Indianapolis, ID, USA) was used to perform a quantitative real-time polymerase chain reaction (qRT-PCR). The primer pair for GLUT2 was 5′-CAG GAC TAT ATT GTG GGC TAA-3′ (forward) and 5′-CTG ATG AAA AGT GCC AAG T-3′ (reverse); for GLUT5, 5′-ACC GTG TCC ATG TTT CCA TT-3′ (forward) and 5′-ATT AAG ATC GCA GGC ACG AT-3′ (reverse); and for SGLT1, 5′-TGG CAA TCA CTG CCC TTT A-3′ (forward) and 5′-TGC AAG GTG TCC GTG TAA AT-3′ (reverse). The amount of GLUT2, GLUT5, and SGLT1 mRNA was normalized to the amount of mRNA of the housekeeping gene, human β-actin. The primer pair for β-actin was: 5′-AGA GCC TCG CCT TTG CCG AT-3′ (forward) and 5′-CCA TCA CGC CCT GGT GCC T-3′ (reverse). Cycling conditions for human GLUT2, GLUT5, SGLT1, and β-actin amplification were the same as those described by Peixoto et al. [28]. Data were analyzed using LightCycler^®^ 96 SW 1.1 analysis software (Roche, Mannheim, Germany), and the results were analyzed by the Ct method [29]. The β-actin mRNA expression levels were not affected by cell treatment.

#### 2.5.7. Total Protein Determination

The protein content of cell monolayers was determined using human serum albumin as standard, according to the method of Bradford [30].

### 2.6. Statistical Analysis

For chemical analyses, the results were expressed as mean ± standard deviation (*n* = 3), and statistical analyses were performed using IBM SPSS 26.0 for Windows (SPSS Inc., Chicago, IL, USA). One-way ANOVA was used to assess significant differences between samples, followed by Tukey’s HSD post hoc test to make pairwise comparisons between the means. For cellular experiments, data were expressed as means ± standard error of the mean (*n* = 6–9), using the GraphPad Prism version 7.0 software (San Diego, CA, USA). In this case, statistical differences between the two groups were evaluated by Student’s *t*-test. The level of significance for all tests (*p*) was 0.05.

## 3. Results and Discussion

### 3.1. CGA Profile and Caffeine Contents of the Different Extracts

Table 1 details the composition of green coffee, roasted coffee, and coffee silverskin extracts regarding their CGA profile and caffeine contents.

The green coffee extract showed the richest CGA profile, as expected, presenting significantly higher amounts (*p* < 0.05) of all the individual CGA than the roasted coffee and silverskin extracts. In fact, it is well known that during the roasting process, these compounds are degraded or transformed into lactones and melanoidins [6,31,32,33,34]. In contrast, roasted coffee extract presented significantly higher amounts of caffeine than the green extracts, which might be explained due to the weight loss of coffee beans during roasting, which was also reported in previous studies [35,36,37]. In general, the amounts of caffeine and CGA found for both green and roasted coffee extracts were similar to those previously reported for aqueous extracts of the respective matrices [38,39,40]. Although in significantly lower amounts than in green and roasted coffees, the silverskin extract also contained CGA, as well as caffeine. In comparison with other studies, the amounts found in our silverskin extract were higher than those reported by Panusa et al. [41] for a robusta coffee silverskin aqueous extract (caffeine: 3.75 mg/g; CGA (expressed as 5-CQA equivalents): 1.25 mg/g), but the amounts of 5-CQA were relatively similar to those described by Puga et al. [26], where different aqueous silverskin extracts were also prepared by an ultrasound extraction method. Compared to a previous study in which we characterized silverskin extracts from a mixture of arabica and robusta silverskin (kindly provided by a national coffee company, as their major by-product) [28], in the present study, the obtained values were higher (32.8 against 27.7 mg/g, for caffeine, and 8.09 versus 4.19 mg/g, for total CGA). This was expected, since we focus our study only on silverskin from robusta coffee, which is known to have higher levels of these secondary metabolites [41,42]. However, our results were, in general, lower than those found in other studies using solvents other than water [9,11,33,43]. Nonetheless, considering that we aim to obtain a sustainable extract that can be used without restrictions in the food area, particularly in the development of a functional food or beverage rich in CGA, the amounts obtained were quite satisfactory and, as mentioned above, were comparable to those obtained from other aqueous extracts. Among all the CGA detected, 5-CQA was the main CGA found in all extracts (representing around 33–51% of total CGA). Furthermore, of all compounds that were investigated in this study, caffeine and 5-CQA were the most representative compounds detected and quantified.

### 3.2. Effects on Cell Viability and Culture Mass

**Figure 1:**
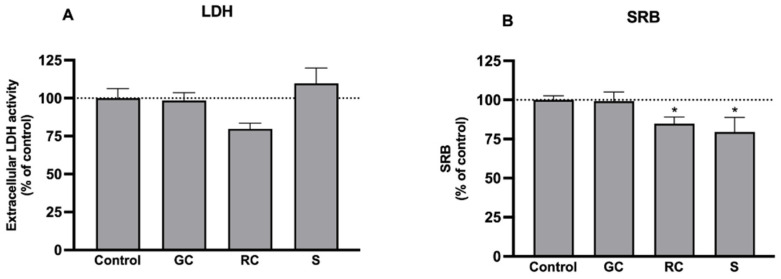
Effect of green coffee (GC), roasted coffee (RC), and coffee silverskin (S) extracts (1 mg/mL) on: (**A**) Caco-2 cell viability, determined by quantification of extracellular LDH activity, and (**B**) culture growth, determined with the SRB assay. Cells were cultured for 24 h in the presence of GC, RC, and S extracts (*n* = 4–10) or the respective solvent (control; *n* = 8–10). The results are shown as arithmetic means ± SEM. *, significantly different from control (*p* < 0.05; Student’s *t*-test).

In order to determine whether the extracts affect cell viability and proliferation, LDH (cell viability) and the SRB (culture mass) assays were performed. At 1 mg/mL, the extracts were not cytotoxic (Figure 1A). Nonetheless, through the SRB assay, we found that the culture mass was slightly reduced by roasted coffee and silverskin extracts (by 15–20%), compatible with a modest antiproliferative effect (Figure 1B). Therefore, we decided to test these extracts in concentrations up to 1 mg/mL (which are not cytotoxic) in the following experiments. In future studies, it would be interesting to better address this effect to explore other beneficial properties of the extracts—particularly the antitumoral effects—in addition to those studied herein.

### 3.3. Effects on the Uptake of ^3^H-DG

Coffee and its by-products, such as silverskin, are being increasingly studied due to their richness in bioactive compounds, and it is known that some of them are able to inhibit intestinal sugar absorption [18,19,20,21,22,23]. In the present study, the effect of coffee silverskin on the uptake of glucose (^3^H-DG) by Caco-2 cells was explored and compared with that of green and roasted coffee.

**Figure 2:**
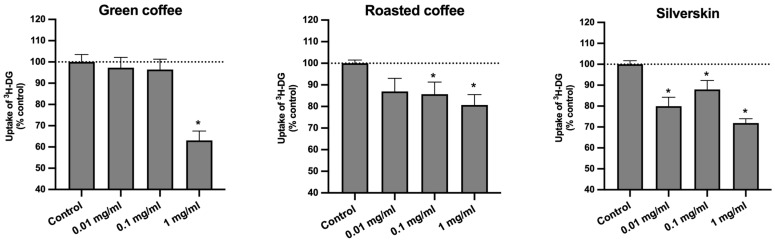
Effect of green coffee, roasted coffee, and coffee silverskin extracts on the uptake of ^3^H-DG by Caco-2 cells. Caco-2 cells were cultured for 24 h in the presence of different extract concentrations (*n* = 6–9) or the respective solvent (control; *n* = 7–9). Uptake was measured by incubating Caco-2 cells at 37 °C with 10 nM ^3^H-DG for 6 min. Results are expressed as means ± SEM. *, significantly different from control (*p* < 0.05; Student’s *t*-test).

As shown in Figure 2, green coffee, roasted coffee, and coffee silverskin extracts were able to significantly reduce the absorption of ^3^H-DG by Caco-2 cells.

It is important to note that green coffee at 1 mg/mL was able to reduce the uptake of ^3^H-DG by almost 40%. These results are quite interesting, since CGA were detected in our study by HPLC-DAD in significantly higher concentrations in green coffee extracts (Table 1). These compounds are widely recognized as the main phenolic compounds in coffee and its by-products [11], to which the modulation of glucose metabolism (including the inhibition of intestinal glucose transport) has already been attributed [18,19,20,21,22,23], thus emphasizing the relevance of these results. However, no significant difference (*p* > 0.05) was found between the effect of green coffee and silverskin at 1 mg/mL on the uptake of ^3^H-DG, but a significantly smaller effect (*p* < 0.05) of roasted coffee extract (1 mg/mL) was observed when compared with green coffee extract at the same concentration. This observation is unexpected, because silverskin extracts presented lower amounts of total CGA (8.1 mg/g of lyophilized extract) when compared to green and roasted coffee extracts (253.1 and 79.5 mg/g of lyophilized extract, respectively).

This led us to formulate various hypotheses. On the one hand, it is possible that, although smaller, the amount of CGA found in silverskin extracts is still able to inhibit the uptake of ^3^H-DG. This is based on the fact that several studies indicated that even small high amounts of polyphenolic compounds are sufficient to elicit a biological response [12,34]. On the other hand, it is possible that the different amounts of caffeine found in the three extracts also affected the absorption of ^3^H-DG and, additionally, may have interacted with CGA. While the amount of CGA in the green coffee extract was significantly higher than the amount of caffeine (253.1 mg CGA and 51.7 mg caffeine/g of lyophilized extract), an opposite relationship between these compounds was found in the silverskin extract (8.1 mg CGA and 32.8 mg caffeine/g of lyophilized extract), and in roasted coffee extracts (which presented the highest amount of caffeine of all extracts), the amounts of CGA and caffeine were rather similar (79.5 mg CGA and 60.2 mg caffeine/g of lyophilized extract). Considering the ratio between CGA and caffeine for the different extracts, green coffee presented 83% CGA/17% caffeine, roasted coffee presented 57% CGA/43% caffeine, and silverskin presented 20% CGA/80% caffeine. As can be noted, although green coffee extracts possess high amounts of caffeine, the relative percentage of this compound compared to CGA is rather small, whereas in the roasted extract, both a high amount and a high relative percentage of caffeine were observed. In contrast, in silverskin extracts, although a high relative percentage of caffeine was found, the amount was not as high as those found in the other two extracts. Thus, it can be suggested that, depending on the proportion of CGA and caffeine present in the extract, as well as on the corresponding amounts of caffeine, this compound may act synergistically or antagonistically with CGA for the inhibition of intestinal glucose transporters activity and/or expression. In this context, it is noteworthy that some studies have tried to find a relationship between caffeine and CGA content in coffee and its effects on glucose metabolism, but, as far as we know, no consensus has yet been reached [32,44,45,46].

**Figure 3:**
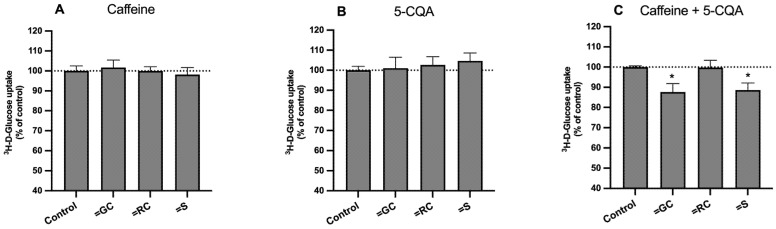
Effect of (**A**) caffeine, (**B**) 5-caffeoylquinic acid (5-CQA), and (**C**) a mixture of both in the same concentrations present in the respective extracts (GC, green coffee; RC, roasted coffee; S, silverskin) on the uptake of ^3^H-DG by Caco-2 cells. Caco-2 cells were cultured for 24 h in the presence of the standards (*n* = 6–7) or the respective solvent (control; *n* = 6–7). Uptake was measured by incubating the cells at 37 °C with 10 nM ^3^H-DG for 6 min. Results are expressed as means ± SEM. *, significantly different from control (*p* < 0.05; Student’s *t*-test).

Therefore, we decided to investigate the putative involvement of caffeine and CGA regarding the effect of the extracts on the uptake of ^3^H-DG and the possible interactions between these two compounds. For this, we evaluated the effects of caffeine and 5-CQA (the main CGA found in all samples) on the uptake of ^3^H-DG by Caco-2 cells, at the concentrations present in 1 mg/mL of the corresponding extracts (caffeine: 5.17 × 10^−2^ mg/mL for green coffee, 6.02 × 10^−2^ mg/mL for roasted coffee, and 3.28 × 10^−2^ mg/mL for silverskin; 5-CQA: 0.13 mg/mL from green coffee, 2.66 × 10^−2^ mg/mL for roasted coffee, and 0.35 × 10^−2^ mg/mL for silverskin). These concentrations were chosen because they had the most marked effects on sugar uptake and did not interfere with cell viability (Figure 1). These bioactive substances were tested separately and together (thus mimicking each extract), and the results are presented in Figure 3.

Interestingly, although neither compound was individually able to affect the uptake of ^3^H-DG, the combinations of caffeine and 5-CQA, as found in green coffee and silverskin extracts (but not in roasted coffee), caused a significant inhibition (≅12% of control) (Figure 3C). The amount of cell protein was not changed by any of the treatments (results not shown), which suggests that the number of cells was not significantly changed. Thus, a synergic effect between caffeine and 5-CQA appears evident. These results corroborate the previously raised hypothesis about the importance of not only the amount of CGA and caffeine, but also their relative proportions in the extracts. These results also suggest that other compounds, beyond 5-CQA and caffeine, may also be involved in the inhibition of ^3^H-DG uptake by all the extracts, since higher reductions were found with the extracts when compared with caffeine and/or CGA. For example, besides other CGA, some CGA derivatives, such as CGA lactones (CGL) and melanoidins, might also be present in our samples, and these compounds have already been described to possess bioactive properties, namely positive effects on glucose metabolism [3,31]. Thus, in further studies, the presence of these and other non-CGA compounds previously described for coffee (e.g., trigonelline, cafestol, isoflavones, etc. [3,47]) should also be investigated, as well as their effects on the absorption of glucose.

### 3.4. Effect on the Uptake of ^14^C-FRU

**Figure 4:**
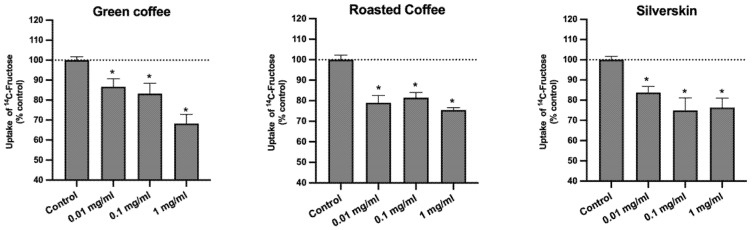
Effect of green coffee, roasted coffee, and coffee silverskin extracts on the uptake of ^14^C-FRU by Caco-2 cells. Caco-2 cells were cultured for 24 h in the presence of different extract concentrations (*n* = 9) or the respective solvent (control; *n* = 9). Uptake was measured by incubating Caco-2 cells at 37 °C with 100 nM ^14^C-FRU for 6 min. Results are expressed as means ± SEM. *, significantly different from control (*p* < 0.05; Student’s *t*-test).

Considering that the intake of fructose is also positively associated with the risk of development of metabolic syndrome [14,15], we also evaluated the effect of the extracts on the uptake of ^14^C-FRU by Caco-2 cells. We verified that all the samples significantly reduced the absorption of this sugar (Figure 4).

Curiously, the inhibitory effect of the three extracts was very similar (no significant differences found, *p* > 0.05). So, similarly to what was found for the uptake of ^3^H-DG, coffee silverskin has proven to be as effective as green coffee and roasted coffee in reducing the uptake of ^14^C-FRU, despite possessing lower amounts of CGA and caffeine. Nevertheless, contrary to what was observed for the uptake of ^3^H-DG, the roasting process does not seem to interfere with the effect of coffee upon absorption of ^14^C-FRU, since similar reductions were found in cells treated with green and roasted coffee extracts.

**Figure 5:**
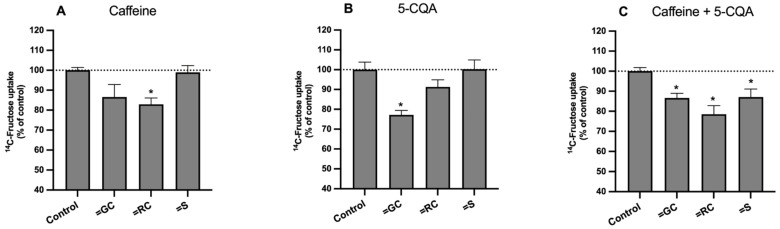
Effect of (**A**) caffeine, (**B**) 5-caffeoylquinic acid (5-CQA), and (**C**) a mixture of both (in the same concentrations present in the respective extracts (GC, green coffee; RC, roasted coffee; S, silverskin) on the uptake of ^14^C-FRU by Caco-2 cells. Caco-2 cells were cultured for 24 h in the presence of the standards (*n* = 6–9) or the respective solvent (control; *n* = 6–9). Uptake was measured by incubating the cells at 37 °C with 100 nM ^14^C-FRU for 6 min. Results are expressed as means ± SEM. *, Significantly different from control (*p* < 0.05; Student’s *t*-test).

In parallel to what was performed for the uptake of ^3^H-DG, the effects of caffeine and 5-CQA, individually and in combination (in the concentrations found in each extract), were also studied to evaluate the potential involvement of these compounds on the effect of the extracts on the uptake of ^14^C-FRU, and to explore possible interactions between them (Figure 5). The results showed that caffeine and 5-CQA (at the concentrations present in roasted and green coffee extracts, respectively) significantly decreased the uptake of ^14^C-FRU by Caco-2 cells (Figure 5A,B). Finally, when combined in the same proportions as in the extracts (Figure 5C), the mixtures “caffeine + 5-CQA” were able to reduce the uptake of ^14^C-FRU (by 14%, 22%, and 13%, respectively) by a similar degree as was observed with green coffee, roasted coffee, and silverskin (Figure 4). As already noted, the amount of cell protein was not changed by any of the treatments (results not shown), suggesting that the number cells was not significantly changed. These results seem to imply 5-CQA as the main responsible for the reduction in the uptake of ^14^C-FRU found with green coffee extract, and caffeine as the main responsible for the reduction found with the roasted coffee extract. Nonetheless, the synergy between 5-CQA and caffeine was again evident, particularly with the silverskin-mimicking mixture. Moreover, the differences in the effect on the uptake of ^14^C-FRU found with the extracts and with their corresponding combinations of caffeine + 5-CQA demonstrate that other compounds, in addition to these, might also contribute to the inhibitory effect of the extracts upon the uptake of ^14^C-FRU. Thus, as previously mentioned for ^3^H-DG, further studies investigating the effects of compounds other than caffeine and 5-CQA on the uptake of ^14^C-FRU should be carried out.

### 3.5. Effect on on SGLT1, GLUT2, and GLUT5 mRNA Levels

**Figure 6:**
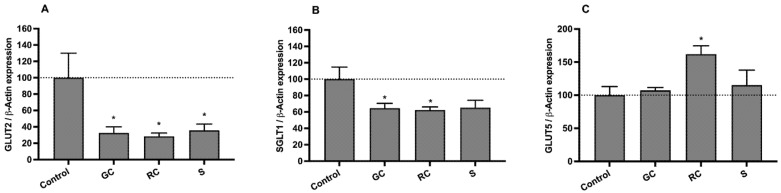
Quantification of mRNA levels of (**A**) facilitative glucose transporter 2 (GLUT2); (**B**) sodium-dependent glucose transporter 1 (SGLT1); and (**C**) facilitative glucose transporter 5 (GLUT5), by RT-qPCR, in Caco-2 cells after a treatment of 24 h with green coffee (GC), roasted coffee (RC), and coffee silverskin (S) extracts (all at 1 mg/mL; *n* = 5–6) or the respective solvent (control, DMSO; *n* = 5–6). The results are shown as the expression of SGLT1, GLUT5, or GLUT2 in relation to β-actin (arithmetic means ± SEM). *, significantly different from control (*p* < 0.05; Student’s *t*-test).

To better characterize the effects of the extracts on the uptake of sugars by Caco-2 cells, the mRNA levels of the main intestinal sugar transporters were also quantified by qRT-PCR. The intestinal absorption of fructose is mediated by two facilitative glucose transporters (GLUTs)—GLUT5 and GLUT2—while the intestinal absorption of glucose is mediated by GLUT2 and by the sodium-glucose cotransporter (SGLT1) [12]. As shown in Figure 6A, chronic (24 h) exposure of Caco-2 cells to the three extracts originated a very marked reduction (*p* < 0.05) in the expression levels of GLUT2 (of 67%, 72%, and 64%, for green, roasted coffee, and silverskin, respectively). Concerning SGLT1, green and roasted coffee extracts were able to significantly decrease (*p* < 0.05) its gene expression (by 36% and 38%, respectively) (Figure 6B). Although with no statistical significance, a decreasing tendency was also found for the coffee silverskin extract. Finally, the green coffee and silverskin extracts did not affect GLUT5 gene expression, but roasted coffee increased it by 62% (Figure 6C).

In summary, these results suggest that the reduction in the uptake of both ^3^H-DG and ^14^C-FRU caused by the three extracts (1 mg/mL) is related to a marked reduction in gene expression of the GLUT2 transporter. This observation is quite encouraging, since GLUT2 has been appointed as the most important pathway for the intestinal absorption of sugars when high glucose and fructose concentrations reach the intestinal lumen (e.g., after a meal) [12,48].

Moreover, by comparing the effects of green coffee and coffee silverskin extracts at 1 mg/mL on the uptake of ^3^H-DG and ^14^C-FRU with their effects on transporter expression levels, we can conclude that the ability of these extracts to markedly decrease gene expression of SGLT1 and GLUT2, but not of GLUT5, might explain the highest reductions in the uptake of ^3^H-DG (which is SGLT1- and GLUT2-mediated) when compared to the uptake of ^14^C-FRU (which is GLUT2- and GLUT5-mediated).

Additionally, we verified that green and roasted coffee extracts caused a similar decrease in the expression levels of SGLT1 and GLUT2, but green coffee induced a greater decrease in the uptake of ^3^H-DG. It is possible that, besides decreasing the expression levels of both glucose intestinal transporters, green coffee may also inhibit these transporters post-transcriptionally, e.g., by directly affecting the activity of SGLT1 and/or GLUT2. Finally, we can also conclude that GLUT5-mediated transport is not vital for the uptake of ^14^C-FRU by Caco-2 cells, because the increase in its gene expression induced by roasted coffee was not accompanied by a significant increase in the uptake of ^14^C-FRU (in comparison with green coffee extract). However, it is worth noting that the increase in GLUT5 gene expression generated by roasted coffee may not have allowed a more pronounced decrease in the uptake of ^14^C-FRU caused by this extract.

In summary, this study shows that green coffee, roasted coffee, and coffee silverskin extracts, which were prepared using a green and cost-effective method (ultrasound-assisted extraction) and are rich in CGA and caffeine, all have an inhibitory effect upon the absorption of glucose and fructose by an intestinal epithelial cell model (Caco-2 cells), which may reveal potential benefits in the context of metabolic syndrome (by reducing the intestinal absorption of sugars). However, further studies are required to assess these effects in an in vivo situation. More particularly, in this study, the green coffee extract was the most efficient inhibitor of ^3^H-DG and ^14^C-FRU uptake by Caco-2 cells, and it was also the extract that contained higher amounts of the mentioned bioactive compounds. Nonetheless, coffee silverskin extract, which presented significantly lower amounts of CGA and caffeine compared to the other extracts, also promoted significant reductions in the uptake of both sugars by Caco-2 cells. This effect was, in general, very similar to those found for green and roasted coffee extracts, and, in the case of ^3^H-DG uptake, it was even higher than that found for roasted coffee. Along with the results obtained for the major compounds identified in the extracts (5-CQA and caffeine, individually and combined), it is suggested that the effects of each extract on the uptake of ^3^H-DG and ^14^C-FRU were dependent on the amounts and proportions of those compounds. Additionally, these findings also demonstrate that the whole extract is much more advantageous than the isolated compounds since, on the one hand, synergic activities were observed between 5-CQA and caffeine, and, on the other hand, the synthetic mixtures presented a smaller effect than the respective original extracts. Furthermore, we must emphasize the inhibitory effect of caffeine and 5-CQA on the uptake of ^3^H-DG and ^14^C-FRU. According to a meta-analysis of interventional studies, the beneficial effects of coffee consumption in reducing intestinal glucose absorption (and thus, blood glucose levels) [49] and on lipid metabolism (an anti-obesity potential) [50] appear to be related to coffee bioactive components such as CGA and caffeine. The findings of the present study clearly show an inhibitory effect of the combination of these two bioactive compounds in the context of sugar intestinal absorption. However, they also suggest that other compounds may also interact with these main bioactives in further inhibiting sugar uptake. In future research, it would be interesting to better characterize these extracts in terms of the presence of other bioactive compounds, such as other CGAs, melanoidins, and CGA lactones, to evaluate their effects on sugar absorption by Caco-2 cells and also to find possible interactions (synergistic, antagonistic, and/or additive) between all compounds that may contribute to the observed effects. Finally, our results support the conclusion that the effects of the extracts on the uptake of ^3^H-DG and ^14^C-FRU result mainly from the inhibition of GLUT2 gene expression, and that a reduction in SGLT1 gene expression also contributed to the reduction in the uptake of ^3^H-DG.

## 4. Conclusions

Overall, all the extracts tested and the synergistic combination between the main bioactives (caffeine and 5-CQA) were found to decrease the uptake of glucose and fructose by the Caco-2 cell line. These results suggest that these extracts may be able to decrease the intestinal absorption of glucose and fructose and therefore, might present beneficial effects on type 2 diabetes and other metabolic disorders. In particular, coffee silverskin appears as a matrix of great interest, since it was shown that, in this context, it is as efficient as green and roasted coffee in producing these effects. Simultaneously, its use (for example, to develop innovative functional food products) will also significantly contribute to answer the needs regarding sustainability and the circular economy of the coffee chain.

## Figures and Tables

**Table 1:** Caffeine and CGA contents (mg/g of freeze-dried extract) of green coffee, roasted coffee, and coffee silverskin extracts prepared by ultrasound-assisted extraction.

	Green Coffee	Roasted Coffee	Coffee Silverskin
Caffeine	51.66 ± 0.92 ^b^	60.22 ± 0.47 ^a^	32.81 ± 1.21 ^c^
5-CQA	129.82 ± 5.02 ^a^	26.57 ± 0.05 ^b^	3.45 ± 0.13 ^c^
4-CQA	38.90 ± 2.09 ^a^	18.00 ± 0.11b ^b^	0.74 ± 0.03 ^c^
3-CQA	24.53 ± 1.50 ^a^	13.78 ± 0.55 ^b^	0.70 ± 0.06 ^c^
5-FQA	52.58 ± 2.77 ^a^	15.75 ± 0.06 ^b^	2.56 ± 0.10 ^c^
4-FQA	7.24 ± 0.38 ^a^	5.39 ± 0.09 ^b^	0.64 ± 0.07 ^c^
Ʃ CGA	253.07	79.49	8.09

Results are expressed as means ± standard deviation (*n* = 3). CQA, caffeoylquinic acid; FQA, feruloylquinic acid; CGA, chlorogenic acids. Within each line, different letters represent significant differences between samples at *p* < 0.05.

## Data Availability

All related data and methods are presented in this paper. Additional inquiries should be addressed to the corresponding author.
